# A Precious Morceau

**Published:** 1851-01

**Authors:** 


*A Precious Morceau.*—The following anecdote is copied from the N. Y. Medical Gazette, and credited by that journal to the *American Homœopathic Journal.* The Editor of the Gazette says the trick has often been repeated in America. [Editor Buffalo Journal.

"In a first-class carriage between Manchester and London, each compartment having its occupant, a gentleman made great contortions, moaned, sobbed convulsively, applied his hand to his stomach, writhed, and exhibited all the signs of suffering. His fellow travelers were alarmed; and he gasped out to the one next to him: "My pocket—a little box—thank you —oh! dear! open—the bottle—marked 'Cocculus,'—two globules—thank you." The globules fairly in his mouth, there came a minute's storm of aggravated pains, groans, moans; then the rigid features relaxed, a benign smile stole over the features, a sigh of relief, then free and frank speech again. 'What was the matter with you, sir?'—'Cramp in the stomach—wonderful the effect of the globules; people should never travel without them.' Then a lecture on Homœopathy; seeing is believing; the fellow-travelers of the man so rapidly relieved of severe suffering, wished to know who he was; then a card is given to each of them, and that the card of a *doctor.*

"It is said to have so chanced, that the same person again seemed to suffer in a like manner, in the course of a journey from London to Manchester, and the same scene would have been repeated again, but one of his fellow-passengers cried out, 'That will do, doctor———, I was present when you did that trick with the little box, some months ago.'"

